# Burnout in Anaesthesiology: A Nationwide Cross-sectional Study of Anaesthesiologists and Residents in Türkiye

**DOI:** 10.4274/TJAR.2026.252258

**Published:** 2026-08-28

**Authors:** Orkun Kopac, Gökhan Sertçakacılar, Yasin Tire, Yutian Luo, Senem Urfalı, Sinem Sarı, Büşra Tok Çekmecelioğlu, Mohammad Khudirat, Sepideh Saroukhani, Ali Alper Kütük, Alparslan Turan

**Affiliations:** 1 McGovern Faculty of Medicine at UTHealth Houston, Center for Outcomes Research and Department of Anaesthesiology, Critical Care and Pain Medicine, Texas, USA; 2 OUTCOMES RESEARCH Consortium®, Texas, United States; 3 University of Health Sciences, Türkiye, Bakırköy Dr. Sadi Konuk Training and Research Hospital, Clinic of Anaesthesiology and Reanimation, İstanbul, Türkiye; 4 Konya City Hospital, Clinic of Anaesthesiology and Reanimation, Konya, Türkiye; 5 Hatay Mustafa Kemal University, Tayfur Ata Sökmen Faculty of Medicine, Department of Anaesthesiology and Reanimation, Hatay, Türkiye; 6 Aydın Adnan Menderes University Training and Research Hospital, Department of Anaesthesiology and Reanimation, Aydın, Türkiye; 7 Cleveland Clinic, Division of Multi-specialty Anaesthesiology, Integrated Hospital Care Institute, Department of Anaesthesiology, Ohio, United States; 8 The University of Texas Health Science Center, McGovern Faculty of Medicine, Division of Clinical and Translational Sciences, Department of Internal Medicine, Texas, United States; 9 The University of Texas Health Science Center, Biostatistics/Epidemiology/Research Design (BERD) component, Center for Clinical and Translational Sciences (CCTS), Texas, United States

**Keywords:** Anaesthesiology, burnout, Maslach Burnout Inventory, Türkiye, wellbeing, workforce

## Abstract

**Objective:**

To determine the prevalence of burnout and to identify factors associated with it among anaesthesiology attendings and residents in Türkiye.

**Methods:**

We conducted a nationwide cross-sectional survey of anaesthesiology departments in Türkiye between September and December 2024. Burnout was assessed using the Maslach Burnout Inventory-Human Services Survey (Turkish version). Burnout syndrome was defined as high emotional exhaustion and depersonalization combined with low personal accomplishment; high-risk status was defined as high emotional exhaustion or depersonalization, regardless of personal accomplishment. Sociodemographic, occupational, and workplace factors were collected, and associations were analysed using univariable and multivariable logistic regression.

**Results:**

Among the 921 respondents (53% residents, 46% attendings; median age 33 years, interquartile range: 29-43; 53% female), 71.6% were at high-risk for burnout, and 36.7% met the criteria for burnout syndrome. Residents comprised a significantly greater proportion of physicians with burnout syndrome (68% vs. 45%; *P* < 0.001) and of those at high-risk for burnout (61% vs. 34%; *P* < 0.001). In the final multivariable model, younger age, female gender, low workplace satisfaction, perceived understaffing, and inadequate personal support were significantly associated with burnout syndrome. The combination of being female and being a resident is associated with higher odds of burnout (interaction *P*=0.034).

**Conclusion:**

The prevalence of burnout among the Turkish anaesthesiology workforce is extremely high and is especially pronounced among female residents and among those who are younger, work in understaffed settings, or experience job dissatisfaction. Targeted, system-level reforms that ensure fair pay, improved staffing, and supportive leadership, coupled with nationwide longitudinal monitoring, are needed to protect clinician well-being and patient safety.

**Main Points**

• In a national survey of 921 anaesthesiology attendings and residents in Türkiye, 71.6% were at high-risk for burnout and 36.7% met the criteria for burnout syndrome.

• Burnout was higher in residents than in attending physicians and decreased with age (6% lower odds per year).

• Female residents were at higher risk.

• Low workplace satisfaction, inadequate staffing, and inadequate support were associated with burnout and intent to resign.

## Introduction

Burnout syndrome, first described by Freudenberger, arises from unmanaged chronic stress.^1^ It is commonly described in the Maslach framework as emotional exhaustion, depersonalization/cynicism, and reduced professional efficacy.^2^ The World Health Organization describes burnout as an occupational phenomenon resulting from chronic workplace stress that has not been successfully managed, characterized by exhaustion, increased mental distance or negativism toward one’s job, and reduced professional efficacy.^3^

Burnout rates vary across medical specialties, with anaesthesiology experiencing particularly high levels due to the specialty’s demanding nature. Given these serious patient safety implications, anaesthesiologists are particularly vulnerable due to their critical role in leading clinical teams under high-pressure conditions. Inadequate staffing further intensifies individual workload and workplace stress.^4^ Unpredictable operating room (OR) schedules, prolonged work hours, and limited time outside the OR restrict opportunities for peer interaction and non-work-related social engagement. Additionally, working in small, shared spaces with few anaesthesiology colleagues and rotating through rapidly changing multidisciplinary teams, which are occasionally marked by conflict, can exacerbate isolation, erode social networks, and leave providers feeling disconnected and undervalued.

Ineffective management of work stress adversely affects physical and emotional health. Physicians report higher burnout rates than the general workforce, reflecting role-specific demands.^5^ The American Society of Anesthesiologists warns that clinician burnout compromises patient safety by increasing medical errors, lowering patient satisfaction, and increasing healthcare costs^6, 7^-and estimates that burnout-driven errors contribute to 210,000-400,000 preventable deaths annually.^8, 9^ Addressing burnout is therefore both a patient-safety imperative and a workforce-sustainability priority.

The prevalence of burnout varies across cultures and countries, but rates in anaesthesiology have been consistently high worldwide. In the United States, Afonso et al.^10^ reported that in March 2020, 59.2% of anaesthesiologists were at high-risk for burnout and 13.8% met clinical criteria, increasing to 67% and 18.9% in November 2022.^11^ High rates have been observed internationally.^12, 13, 14, 15, 16, 17^ Although Turkish data are sparse, existing studies show substantial burnout symptoms,^18^ including pre-pandemic studies in İstanbul^19^ and Central Anatolia,^20^ supporting the need for a comprehensive national evaluation.

This study aims to determine burnout levels among anaesthesiology residents and attendings in Türkiye and identify factors associated with burnout. In Türkiye, residents (trainees) are physicians in specialty training, whereas attendings are fully trained specialist anaesthesiologists (consultant-level).

## Methods

Ethical approval for this study was granted by the University of Health Sciences Türkiye, Bakırköy Dr. Sadi Konuk Training and Research Hospital, Non-Interventional Scientific Research Ethics Committee (approval no: 2024-08-13, date: 19.08.2024). Participation was voluntary and anonymous, and informed consent was implied through the completion of the survey. No identifying personal information was collected. This manuscript adheres to the STROBE guidelines.^21^

### Design

This cross-sectional, correlational study collected data at a single point in time to examine the relationships between various variables and burnout.

### Participants

The target population for this study consisted of attending anaesthesiologists and residents in Türkiye. Participant recruitment took place between September 30 and December 17, 2024. Survey invitations were distributed via the official email list of the Turkish Anaesthesiology and Reanimation Association. To further improve accessibility and participation, the survey was also directly shared with anaesthesiology departments throughout the country.

### Survey Questionnaire

The survey instrument was structured into two primary sections. The first section collected sociodemographic, occupational, and personal information, including age, gender, marital status, number of children, professional rank (resident or attending), type of hospital (university, training, or other), years in practice, weekly work hours, number of night shifts per month, and whether the participant was working in a mandatory service region. The first section also incorporated an optional component assessing job satisfaction, perceived adequacy of workplace and personal support, staffing levels, and intent to resign.

The second section used the 22-item Maslach Burnout Inventory-Human Services Survey (MBI-HSS) Turkish form, a validated tool for assessing burnout.^2, 22^ While the original MBI utilizes a 7-point Likert scale (“never” to “every day”), the Turkish version adopted a 5-point Likert scale (“never” to “all the time”) based on its linguistic and cultural fit, as the original anchors were deemed less intuitive for Turkish respondents.^22^

The MBI evaluates burnout dimensions on a continuum and does not provide universally accepted diagnostic cut-off values. To allow comparison with large prior studies of physician burnout^23^ and with prior anaesthesiology burnout literature using MBI-based categorical definitions^10, 11^ we applied a previously published classification framework to define high-risk for burnout and burnout syndrome. Because the published thresholds were derived from the original 7-point scoring format (0-6) and the Turkish MBI in our study used a 5-point format (0-4), we proportionally rescaled the cut-off values before classification (original cut-off x4/6). Burnout syndrome was defined as high emotional exhaustion (scores of 18-36 in the MBI-HSS emotional exhaustion component), high depersonalization (scores of 7-20 in the MBI-HSS depersonalization component), and low personal accomplishment (scores of 0-22 in the MBI-HSS personal accomplishment component). A high-risk of burnout was defined as high emotional exhaustion or high depersonalization, regardless of the level of personal accomplishment. The detailed numeric definitions of burnout and high-risk for burnout syndrome are noted in Supplementary Metarial 1. Completion of the MBI was a requirement for inclusion in the final dataset.

Preliminary findings from this study were presented at a workshop titled “how to write a manuscript” during the Turkish Anaesthesiology and Reanimation Association annual meeting in december 2024. Workshop participants contributed to the development of a preliminary manuscript and are acknowledged as members of the burnout workgroup in Supplementary Metarial 2.

### Statistical Analysis

Descriptive statistics are reported as frequencies (%) for categorical variables, and as means and standard deviations or medians and interquartile ranges (IQRs) for continuous variables, according to their distribution. Age was analysed as a continuous variable; all other questionnaire variables were considered categorical. Variables with sparse categories were collapsed or dichotomized a priori (e.g., work hours and number of night shifts per month). Specifically, working hours per week was dichotomized at 50-hour cut-off based on previous reports indicating an average of 49 to 51 working hours per week for anaesthesiologists in Türkiye.^20, 24^ Additional variables (years in the same hospital, years in practice, job satisfaction, and number of children) were grouped into three categories. Univariable associations between each factor and burnout outcomes were assessed using the Wilcoxon rank-sum test for continuous variables and the chi-square test or Fisher’s exact test for categorical variables. The variables that were significantly associated with burnout outcomes in the univariable analysis were included in separate multivariable analyses to determine factors associated with burnout and a high-risk of burnout.^25^ The final multivariable model was fitted following stepwise and purposeful variable selection methods^25^ using *P < *0.1 to obtain the adjusted odds ratios and 95% confidence intervals (CIs) for the associations between each of the factors with burnout syndrome and being at high-risk for burnout as outcomes. The *P* < 0.1 threshold was recommended as a liberal criterion to retain a covariate in purposeful variable selection, as this criterion ensures potentially relevant covariates were not dropped too early in the iterative variable selection process.^25^ As an exploratory analysis, we assessed two-way interactions among predictors—gender, working hours, residency type, and number of children—in relation to the outcome of burnout syndrome, using separate logistic regression models that included interaction terms. If the interaction terms were significant under a liberal criterion with *P < *0.1,^25, 26^ the ORs and 95% CIs were calculated for different levels of the interacting variables in relation to the outcome. All analyses were conducted using R statistical software (version 4.1.1, Posit Software, USA). The results of the final model are interpreted at a 0.05 level of significance.

## Results

### Study Population

Between September 30 and December 17, 2024, the survey was accessed 2,593 times. Of those, 1,143 participants began the survey, and 921 completed the mandatory MBI section (222 were excluded due to incomplete MBI). The questionnaire consisted of two sections: an optional sociodemographic and work-life section and a required MBI. Of the 921 respondents, 486 (53%) were anaesthesiology residents, 427 (46%) were attendings, and 8 (1%) did not select a category; the median age was 33 years (IQR: 29-43), and 53% were female. Participants represented all seven regions, with the majority from the Marmara (34%) and Central Anatolia (24%) regions, which is consistent with regional population densities and the number of healthcare facilities. Most participants were employed at state university hospitals (43%) or state training hospitals (36%) (Table 1 and Supplementary Table S1).

### Univariable analysis of Burnout Syndrome and High-risk for Burnout

Based on responses to the MBI-HSS, emotional exhaustion was experienced by 56.4% of respondents, depersonalization by 57.1%, and a low sense of personal accomplishment by 73.7%. Furthermore, 659 participants (71.6%) were identified as at high-risk for burnout, while 338 (36.7%) met the criteria for burnout syndrome. Residents constituted a higher proportion of physicians with burnout than of those in the no-burnout group (68% vs. 45%, *P* < 0.001). Similarly, a greater proportion of physicians at high-risk for burnout were residents (61% vs. 34%, *P *< 0.001) (Table 2).

Most physicians in both the burnout and high-risk groups were female (58% and 56%, respectively). Burnout was more common among younger and single respondents (median age 31 vs. 35 years for those with and without burnout syndrome, *P *< 0.001; and 32 vs. 39 years for the high-risk and not-high-risk groups, *P *< 0.001). Singles comprised 35% of the burnout group versus 24% of the no-burnout group, and 31% of the high-risk group versus 21% of the not-high-risk group (*P*=0.001 and *P*=0.008, respectively). A higher proportion of physicians with burnout was without children (66% vs. 45%, *P *< 0.001). Working >50 hours/week was more common among those with burnout (74% vs. 58%) and those at high-risk (69% vs. 49%) (both *P *< 0.001). Similarly, job dissatisfaction was more common in the burnout group: 27% reported being “not satisfied at all” with their workplace, compared with 8.8% of those without burnout; conversely, only 17% of those with burnout reported being “satisfied/very satisfied” versus 56% of those without burnout (*P* < 0.001). A higher proportion of participants with burnout had been working in the same hospital for 0-2 years (45% vs. 41%,* P *< 0.001) and for 3-5 years (41% vs. 31%, *P *< 0.001). Perceived inadequate workplace support (34% vs. 16%, *P *< 0.001) and perceived inadequate personal life support (20%vs. 12%, *P *< 0.001) were also significantly associated with burnout. Poor hospital infrastructure (i.e., limitations in facility resources, equipment, and supplies needed to deliver care) was more commonly reported by physicians with burnout; 35% of the burnout group reported suboptimal physical conditions at their institutions, compared with 17% in the non-burnout group (*P *< 0.001). Additionally, a higher proportion of physicians with burnout perceived an insufficient number of anaesthesiology attendings (25% vs. 10%, *P *< 0.001), had insufficient time and financial resources for social activities (21% vs. 9.3%, *P <* 0.001*), *and were “likely” (24% vs. 13%, *P <* 0.001)* or “*very likely” (10%vs. 3.6%,* P *< 0.001) to resign within two years (Table 3).

Among attendings, no significant association was found between simply working at a mandatory service location and burnout; however, those who believed that it negatively affected their work were marginally more likely to meet the criteria for burnout syndrome (65% vs. 38%, *P=*0.066)and significantly more likely to be at high-risk for burnout (60% vs. 21%, *P*=0.006) (Supplementary Table S2). Additionally, while the number of calls taken per month was not significantly associated with burnout syndrome, it was significantly associated with being at high-risk for burnout (*P*=0.030), with more than five calls per month corresponding to a higher proportion at high-risk for burnout (36% vs. 26%).

Among residents, those with 0-5 years of total physician experience (including time before residency) were marginally more likely to meet the criteria for burnout syndrome (66% vs. 58%, *P*=0.059) and significantly morelikely to be at high-risk for burnout (63% vs. 48%, *P*=0.005) (Supplementary Table S3). Additionally, among attendings, total years of physician experience were significantly associated with both burnout syndrome and being at high-risk for burnout. A significantly higher proportion of attendings with burnout or at high-risk for burnout had a total of 6-10 years of physician experience (32% vs. 19%, *P*=0.003; 25% vs. 18%, *P*=0.016, respectively) (Supplementary Table S4).

Among residents, the number of years working at the same hospital was not associated with burnout syndrome or high-risk for burnout(*P*=0.2 and *P*=0.6, respectively). However, among attendings, years working at the same hospital were significantly associated with both outcomes. A higher proportion of attendings with burnout (30% vs. 18%, *P*=0.009) and of attendings at high-risk for burnout had beenworking in the same hospital for 3-5 years. By contrast, fewer attendings with burnout (37% vs. 50%,* P*=0.009) and at high-risk for burnout (42% vs. 55%,* P*=0.016) had worked 6+ years at the same hospital.

Among other factors we investigated, we found no significant association between burnout and either geographic region or hospital type. 

### Multivariable Analysis

In the multivariable models, age remained significantly associated with both burnout syndrome and a high-risk of burnout. Specifically, each additional year of age was associated with a 6% reduction in the odds of burnout syndrome (OR=0.94, 95% CI=0.91-0.96, *P* < 0.001) and an 8% reduction in the odds of being at high-risk for burnout (OR=0.92, 95% CI=0.89-0.95, *P *< 0.001). Working 3-5 years at the same hospital was associated with 60 % higher odds of burnout syndrome compared with working 0-2 years (OR=1.60, 95 % CI=1.10-2.34; *P*=0.015), and 78% higher odds of being at high-risk for burnout (OR=1.78, 95% CI=1.14-2.79, *P*=0.012). Male respondents showed lower odds of experiencing burnout syndrome than female respondents (OR=0.72, 95% CI=0.51-1.01, *P*=0.057). Greater workplace satisfaction was associated with lower odds of burnout. Specifically, compared with those who were “not satisfied at all”, respondents who reported being “satisfied” or “very satisfied” had 71% lower odds of having burnout syndrome (OR=0.29, 95% CI=0.16-0.49, *P*<0.001) and 91% lower oddsof being at high-risk for burnout (OR=0.09, 95% CI=0.03-0.22, *P *< 0.001). Respondents who rated their time and financial situation as “moderately sufficient” for social activities had roughly half the odds of meeting burnout syndrome criteria compared with those who found them “not sufficient” (OR=0.51, 95% CI=0.30-0.87; *P*=0.014). Although the overall effect of this variable narrowly missed conventional significance for burnout syndrome (global *P*=0.051), it was significantly associated with being at high-risk for burnout (global *P*=0.011). Specifically, respondents who rated their financial situation as “moderately sufficient” had 58% lower odds of being at high-risk (OR=0.42, 95% CI=0.19-0.86, *P*=0.023), and those who reported it as “more than sufficient” had 82% lower odds (OR=0.18, 95% CI=0.05-0.57, *P*=0.005) (Table 4).

In addition, strong perceived personal life support was associated with lower odds of both burnout syndrome (OR=0.50, 95% CI=0.26-0.95, *P*=0.035) and high-risk of burnout (OR=0.31, 95% CI=0.14-0.69, *P=0.005) *compared with poor support. The perceived adequacy of anaesthesiology attendings also played a significant role in the multivariable analysis. Respondents who considered the number of attendings “more than sufficient” had significantly lower odds of burnout compared with those who deemed it “not sufficient” (OR=0.41, 95% CI=0.21-0.80, *P*=0.009). Respondents with burnout were more likely to report an intention to resign within two years. Compared with those who were “very unlikely” to resign within the next two years, those who selected “ neutral” had 2.69 times higher odds of experiencing burnout (OR=2.69, 95% CI=1.64-4.46, *P <* 0.001);those who were “likely” to resign had 3.67 times higher odds (OR=3.67, 95% CI=2.10-6.51, *P *< 0.001); and those who were “very likely” to resign had 4 times higher odds (OR=4.00, 95% CI=1.84-8.95, *P *< 0.001).

No subspecialty demonstrated significantly different odds of experiencing burnout syndrome. Neuro-anaesthesiologists were marginally less likely to have burnout (OR=0.41, 95% CI=0.14-1.04, *P*=0.076) and had marginally lower odds of a high-risk status (OR=0.46, 95% CI=0.20-1.02, *P*=0.059) (Table 4) (Supplementary Table 5).

### Subgroup and Interaction Effects

The exploratory subgroup analysis showed a significant gender-by-seniority (attending vs. resident) interaction (*P*=0.034), suggesting that the association between seniority and burnout syndrome varied by gender. Specifically, among attendings, gender was not associated with burnout, whereas among residents males were less likely than females to have burnout syndrome (OR=0.54, 95% CI=0.37-0.78, *P*=0.001), suggesting that being a female resident is associated with higher odds of burnout. Within the same interaction model, when gender groups are examined separately, attendings have significantly lower odds of burnout than residents (OR=0.63, CI=0.41-0.97, *P*=0.038), whereas, females had even lower odds of burnout compared to residents (OR=0.33, CI=0.22-0.50, *P *< 0.001) (Supplementary Table S6).

### Work-life Balance Improvements

Respondents identified several changes that would most benefit their work-life balance: improving physical conditions (60.2%), increasing flexibility in work-hours (51.5%), reducing weekly work-hours (54.6%), increasing the number of vacation days (51.4%), ensuring a sufficient number of anaesthesiology attendings (30.6%), improving workplace culture (58%), increasing support from leadership (62.9%), limiting work-related communication outside work-hours (23.7%), improving salary (79.9%), providing soft skills training (26.7%), assisting with child/elder care (18%), and improving the efficiency of electronic medical records (34.4%) (Table 5).

## Discussion

This national study highlights the scale of burnout within the Turkish anaesthesiology workforce. Overall, 71.6% were at high-risk, and 37% met the criteria for burnout syndrome. Burnout was more common in residents than in attendings: roughly four in five residents were at high-risk, and nearly half met the burnout criteria, compared with about three in five and one in four, respectively, among attendings. These rates generally exceed those reported elsewhere. Reported attending burnout rates were lower in Italy (10.2%),^17^ France (24%),^13^ Poland (18%).^14^ Similarly, burnout rates of 25% among United Kingdom trainees,^27^ and 18% overall in the Netherlands^28^ are below our estimates. Even countries with heavy workload pressures, such as Portugal^15^ and Egypt,^12^ report lower rates. China reported a 69% burnout rate, but this figure is likely to reflect the use of lower thresholds for emotional exhaustion and depersonalization scores.^4^ In the United State (U.S.) attending burnout rose from 13.8% in 2020^10^ to 18.9% in 2022^11^ post-pandemic–still well below the 37% observed in our study.

Previous studies from Türkiye provide useful context, but they are mostly single-center or regional, or they focus on selected groups (e.g., residents); some findings differ from those in our cohort. These studies likewise show a substantial burden of burnout and occupational stress among anaesthesiology physicians.^19, 20, 29, 30, 31^ For example, Bıçak and Çelik^29^ found no gender differences in burnout or job satisfaction among specialists in Diyarbakır, whereas burnout risk in our cohort was more pronounced in female residents. In trainee studies, Büget et al.^30^ highlighted workload, training conditions, and the working environment as key stressors, and Abut et al.^19^reported associations with gender and marital status that differed from our findings.We observed no subspecialty differences, unlike reports from algology and anaesthesiology settings.^20^ Recent nationwide data also suggested lower burnout among anaesthesiologists with children, and higher imposter syndrome levels among women.^31^ We did not find an association between having children and burnout. Overall, these discrepancies indicate heterogeneity across Turkish studies, likely reflecting differences in populations, institutions, sample sizes, and measurement and analytic approaches. Smaller studies may also be more sensitive to sampling variation, and associations observed in such studies may not persist after multivariable adjustment in larger cohorts.

We found that Türkiye recorded the highest rate of low personal accomplishment (73.7%) and one of the highest depersonalization scores (57.1%) among published anaesthesiology cohorts. By comparison, Portugal reported 44.8% low personal accomplishment and 90.9% depersonalization,^15^ while Egypt reported 58.2% and 56.1%, respectively.^12^ While the depersonalization in Egypt is comparable to ours, low personal accomplishment in our cohort remains notably higher. Consistent with this, a pan-European workforce survey ranked Türkiye first for exhaustion,^32, 33^ supporting the need for system-level reform rather than relying on individual resilience alone.

Younger physicians had higher odds of burnout syndrome with each additional year of age was associated with a 6% reduction in the odds of burnout, mirroring findings from the U.S,^10, 11^ Portuguese attendings^15^ and İstanbul trainees.^19^ Total years in practice were not associated with burnout. Instead, tenure at a single institution emerged as an associated factor, those with 3-5 years at the same hospital had higher odds of burnout syndrome (OR=1.60) and high-risk status (OR=1.78) than those with ≤2 years, whereas staff with ≥6 years did not. This resembles the mid-career peak described by Dyrbye et al.^34^ whereas our data suggests a mid-tenure peak within one institution. To our knowledge, no study of anaesthesiology burnout has stratified years of practice into finer brackets or demonstrated a significant continuous association; nor has any broader medical study evaluated single-institution tenure, which would add a novel perspective. Mid-tenure clinicians may experience unique pressures: evolving roles, increasing clinical and administrative responsibilities, plateauing promotion opportunities, and an institutional culture warranting further investigation.

Türkiye’s standard 40-45 hours/week refers to scheduled daytime work rather than an upper limit and does not include night-call duties, which are regulated separately in specialty training: night-call is scheduled ≥3 days apart, ≤8/month, and post-call trainees have no clinical duty the next day. As a result, “weekly work-hours” may not capture total workload, complicating comparisons with systems where total duty time is explicitly capped (e.g., the European Working Time Directive, 48 hours/week on average) and helping explain why workload can, in practice, approach or exceed the U.S. residency limit (80 hours/week averaged) when night call is frequent.^34, 35, 36^ In this context, we found no association between work-hours and burnout, consistent with reports from Portugal,^15^ France,^37^ and UK trainee cohort.^27^ Conversely, Li et al.^4^ found higher burnout among those working >60 hours/week in China, and Afonso’s U.S. data showed that >40 hours/week were associated with burnout and high-risk status.^11^ Overall, these mixed findings suggest that the work-hours-burnout relationship is context-dependent, shaped by how hours are defined (scheduled hours vs. total workload, including night call) and by organizational factors that affect recovery time and staffing support.

Residents reported higher burnout rates than attendings, mirroring data from the U.S.^11, 38^ and an Egyptian academic center,^12^ but contrasting with Dutch^28^ and Chinese^4^ studies where junior attendings had higher rates. This pattern may reflect the demands of residency, including long hours, frequent on-call duties, a steep learning curve, and limited autonomy. Among residents, we observed lower odds of burnout in male residents, similar to U.S. data.^38^ Female residents may face added pressures from more emotionally demanding patients and family interactions, difficulty declining additional tasks, and completing domestic responsibilities, which can reduce the time available for rest and recovery. By contrast, female attendings may be buffered by greater autonomy, established professional networks, and institutional support, which could attenuate gender differences seen during training. Mitigating these stressors requires equitable task assignments, robust mentorship, and flexible scheduling to support work-life balance.

Staffing shortages and workplace dissatisfaction showed the strongest associations with burnout. One-third of clinicians experiencing burnout reported a high likelihood of leaving their positions, which represents an alarming threat amid ongoing staff shortages. 80% of respondents called for higher salaries, and over half identified needs such as better leadership engagement, improved working conditions and workplace culture, greater scheduling flexibility, and more vacation time. In China, where compensation is also relatively low, insufficient pay has been associated with burnout.^4^ Consistent with U.S. studies,^38, 39^ respondents reporting adequate income and time for personal life had roughly half the odds of burnout, underscoring the protective role of work-life balance and fair compensation. Perceived personal support was the only non-work-related factor associated with lower odds of burnout, reinforcing that organizational factors outweigh individual ones, as also noted in U.S. studies.^10, 11^

The strengths of our study include the large, diverse national sample and the use of the validated Turkish version of the MBI, enabling comparisons with prior studies. To our knowledge, this is the first nationwide study to estimate burnout prevalence and explore associated factors among Turkish anaesthesiology attendings and residents.

### Study Limitations

This study has limitations. The cross-sectional design and self-reported data limit causal inference and may introduce reporting bias. Voluntary participation raises the possibility of selection bias and non-response bias. Individuals with higher burnout or stronger views may have been more likely to respond, inflating estimates of burnout prevalence, whereas those with severe burnout may have been less able or willing to participate, deflating estimates of burnout prevalence, respectively. Because we lack data on non-responders and cannot calculate response rates, the direction and magnitude of any bias are uncertain. Comparisons with prior Turkish studies should be interpreted cautiously due to differences in study populations, settings, sample sizes, and measurement instruments.

The classification of burnout is another limitation. Although the Turkish MBI has been psychometrically validated, commonly used categorical cut-offs vary across studies and are not clinically or culturally validated diagnostic thresholds.^40^ We therefore applied a widely used MBI-based classification framework from the physician literature and rescaled the original 7-point thresholds to the Turkish 5-point format for comparability.^10, 11, 40^ These adapted cut-offs were not re-validated in our Turkish sample, and any misclassification is most likely near the thresholds.

## Conclusion

Turkish anaesthesiologists—particularly younger clinicians, mid-tenure staff, and female residents—face among the highest levels of burnout worldwide, associated with systemic stressors such as heavy workloads, understaffing, and inadequate compensation. Leadership must systematically address these pressures by translating them into concrete, monitored action plans designed to measurably improve clinician well-being and, ultimately, patient care.

## Ethics

**Ethics Committee Approval:** Ethical approval for this study was granted by the University of Health Sciences Türkiye, Bakırköy Dr. Sadi Konuk Training and Research Hospital, Non-Interventional Scientific Research Ethics Committee (approval no: 2024-08-13, date: 19.08.2024).

**Informed Consent:** Participation was voluntary and anonymous, and informed consent was implied through the completion of the survey.

## Supplementary Materials

Supplementary Tables https://d2v96fxpocvxx.cloudfront.net/bda9171a-fae8-4995-8276-2138323f1e16/content-images/16a94bde-dd76-4585-a564-e2c50d65fb23.pdf

Supplementary Metarial 1 https://d2v96fxpocvxx.cloudfront.net/353d5d5c-62fa-4c66-b2ed-311e6d47e107/content-images/2921948b-d444-4948-abe6-d8725ac62638.pdf

Supplementary Metarial 2 https://d2v96fxpocvxx.cloudfront.net/353d5d5c-62fa-4c66-b2ed-311e6d47e107/content-images/2151a14e-d1f9-442f-9547-9a0593767ff4.pdf

## Figures and Tables

**Table 1. Demographics:** 

**Characteristic**	**n = 921^1^**
Geographic region	
Aegean Region	105 (12%)
Black Sea Region	57 (6.3%)
Central Anatolia Region	213 (24%)
Eastern Anatolia Region	116 (13%)
Marmara Region	308 (34%)
Mediterranean Region	74 (8.2%)
Southeastern Anatolia Region	28 (3.1%)
Age (yr)	33 (29.43)
Gender	
Female	489 (53%)
Male	427 (47%)
Marital status	-
Single	256 (28%)
Married	620 (68%)
Divorced or widowed	34 (3.7%)
Number of status	
0	478 (53%)
1	209 (23%)
>2	222 (24.3%)
Seniority Status	
Resident	486 (53%)
Attending	427 (46.4%)
Years working as a physician	
0-5 years	306 (33%)
6-10 years	256 (28%)
11-20 years	158 (17%)
21+ years	197 (21.7%)
Years working in the same hospital	
0-2 years	392 (43%)
3-5 years	317 (35%)
6-11 years	105 (11.4%)
12+ years	103 (11.1%)

^1^:n (%); Median (Q1, Q3)

**Table 2. Summary of Questions Based on all Respondents (Q1-Q25, except Q8-Q12) by Burnout Groups:** 

**-**	**Burnout syndrome**			**High-risk for burnout**		
**Characteristic**	**Yes**n = 338^1^	**No**n = 583^1^	***P*** **value** ^2^	**Yes**n = 659^1^	**No**n = 262^1^	***P*** **value** ^2^
Age (yr)	31 (29, 35)	35 (30, 47)	<0.001	32 (29, 39)	39 (32, 50)	<0.001
Gender	-	-	0.038	-	-	0.014
Female	195 (58%)	294 (51%)	-	367 (56%)	122 (47%)	-
Male	142 (42%)	285 (49%)	-	289 (44%)	138 (53%)	-
Marital status	-	-	0.001	-	-	0.008
Single	116 (35%)	140 (24%)	-	201 (31%)	55 (21%)	-
Married	208 (63%)	412 (71%)	-	424 (65%)	196 (76%)	-
Divorced/widowed	8 (2.4%)	26 (4.5%)	-	26 (4.0%)	8 (3.1%)	-
Number of children	-	-	<0.001	-	-	<0.001
0	220 (66%)	258 (45%)	-	389 (60%)	89 (34%)	-
1	58 (17%)	151 (26%)	-	140 (22%)	69 (27%)	-
≥2	54 (16%)	168 (29%)	-	121 (19%)	101 (39%)	-
Seniority status	-	-	<0.001	-	-	<0.001
Resident	227 (68%)	259 (45%)	-	399 (61%)	87 (34%)	-
Attendings	109 (32%)	318 (55%)	-	255 (39%)	172 (66%)	-
Years working as a physician	-	-	<0.001	-	-	<0.001
0-5 years	153 (45%)	153 (26%)	-	255 (39%)	51 (20%)	-
6-10 years	104 (31%)	152 (26%)	-	199 (30%)	57 (22%)	-
10+ years	80 (24%)	275 (47%)	-	202 (31%)	153 (59%)	-
Years working in the same hospital	-	-	<0.001	-	-	<0.001
0-2 years	152 (45%)	240 (41%)	-	292 (44%)	100 (39%)	-
3-5 years	139 (41%)	178 (31%)	-	252 (38%)	65 (25%)	-
6+ years	47 (14%)	161 (28%)	-	114 (17%)	94 (36%)	-
Work hours per week	-	-	<0.001	-	-	<0.001
<50 hours	89 (26%)	246 (42%)	-	202 (31%)	133 (51%)	-
>50 hours	249 (74%)	334 (58%)	-	455 (69%)	128 (49%)	-
Satisfaction with workplace	-	-	<0.001	-	-	<0.001
Not satisfied at all	90 (27%)	51 (8.8%)	-	134 (20%)	7 (2.7%)	-
Somewhat satisfied	190 (56%)	205 (35%)	-	333 (51%)	62 (24%)	-
Satisfied/very satisfied	57 (17%)	323 (56%)	-	188 (29%)	192 (74%)	-

^1^:Median (Q1, Q3); n (%); ^2^: Wilcoxon rank sum test; Pearson’s chi-squared test

**Table 3. Summary of Questions Based on All Respondents by Burnout Groups:** 

**-**	**Burnout syndrome**			**High-risk for burnout**		
**Characteristic**	**Yes**n = 338^1^	**No**n = 583^1^	***P*** **value** ^2^	**Yes**n = 659^1^	**No**n = 262^1^	***P*** **value** ^2^
Support at work	-	-	<0.001	-	-	<0.001
Don’t feel supported at all	116 (34%)	95 (16%)	-	190 (29%)	21 (8.0%)	-
Feel a little supported	161 (48%)	241 (41%)	-	313 (48%)	89 (34%)	-
Feel supported	60 (18%)	204 (35%)	-	142 (22%)	122 (47%)	-
Feel very supported	1 (0.3%)	41 (7.1%)	-	13 (2.0%)	29 (11%)	-
Support outside hospital	-	-	<0.001	-	-	<0.001
Don’t feel supported at all	66 (20%)	69 (12%)	-	120 (18%)	15 (5.8%)	-
Feel a little supported	138 (41%)	181 (31%)	-	251 (38%)	68 (26%)	-
Feel supported	99 (29%)	231 (40%)	-	204 (31%)	126 (49%)	-
Feel very supported	34 (10%)	96 (17%)	-	80 (12%)	50 (19%)	-
Sufficient anaesthesiologist staffing	-	-	<0.001	-	-	<0.001
Not sufficient	83 (25%)	58 (10%)	-	122 (19%)	19 (7.3%)	-
Some shortcomings but manageable	131 (39%)	169 (29%)	-	229 (35%)	71 (27%)	-
Sufficient	98 (29%)	251 (43%)	-	233 (36%)	116 (44%)	-
More than sufficient	24 (7.1%)	102 (18%)	-	71 (11%)	55 (21%)	-
Adequate physical conditions	-	-	<0.001	-	-	<0.001
Not sufficient	118 (35%)	100 (17%)	-	186 (28%)	32 (12%)	-
Some shortcomings but manageable	157 (47%)	261 (45%)	-	316 (48%)	102 (39%)	-
Sufficient	49 (15%)	170 (29%)	-	123 (19%)	96 (37%)	-
More than sufficient	13 (3.9%)	50 (8.6%)	-	32 (4.9%)	31 (12%)	-
Consider changing hospital	245 (73%)	236 (41%)	<0.001	411 (63%)	70 (27%)	<0.001
Probability of resigning within 2 years	-	-	<0.001	-	-	<0.001
Very unlikely	40 (12%)	191 (33%)	-	125 (19%)	106 (41%)	-
Unlikely	67 (20%)	166 (29%)	-	155 (24%)	78 (30%)	-
Neutral	116 (34%)	126 (22%)	-	196 (30%)	46 (18%)	-
Likely	80 (24%)	76 (13%)	-	129 (20%)	27 (10%)	-
Very likely	35 (10%)	21 (3.6%)	-	52 (7.9%)	4 (1.5%)	-
Time and finances for social activities	-	-	<0.001	-	-	<0.001
Not sufficient	70 (21%)	54 (9.3%)	-	113 (17%)	11 (4.2%)	-
Slightly sufficient	170 (50%)	213 (37%)	-	306 (47%)	77 (30%)	-
Moderately sufficient	90 (27%)	279 (48%)	-	226 (34%)	143 (55%)	-
More than sufficient	8 (2.4%)	33 (5.7%)	-	13 (2.0%)	28 (11%)	-

^1^:Median (Q1, Q3); n (%); ^2^:Wilcoxon rank sum test; Pearson’s chi-squared test

**Table 4. Multivariable Analysis of Burnout Syndrome and High-Risk for Burnout Syndrome, after Stepwise Model Building Process and Purposeful Variable Selection:** 

**-**	**Burnout syndrome**			**High-risk for burnout**		
**Characteristic**	**OR** ^1^	**95%** **CI**	***P*** **value**	**OR**	**95%** **CI**	***P*** **value**
Age (year)	0.94	0.91, 0.96	<0.001	0.92	0.89, 0.95	<0.001
Gender	-	-	-	-	-	-
Female	—	—	-	-	-	-
Male	0.72	0.51, 1.01	0.057	-	-	-
Years working in the same hospital	-	-	0.043	-	-	0.029
0-2 years	—	—	-	—	—	-
3-5 years	1.60	1.10, 2.34	0.015	1.78	1.14, 2.79	0.012
6+ years	1.51	0.80, 2.87	0.2	1.65	0.90, 3.08	0.11
Satisfaction with workplace	-	-	<0.001	-	-	<0.001
Not satisfied at all	—	—	-	—	—	-
Somewhat satisfied	0.71	0.44, 1.13	0.2	0.22	0.07, 0.57	0.004
Satisfied/very satisfied	0.29	0.16, 0.49	<0.001	0.09	0.03, 0.22	<0.001
Support outside hospital	-	-	0.014	-	-	0.001
Don’t feel supported at all	—	—	-	—	—	-
Feel a little supported	1.19	0.72, 1.97	0.5	0.61	0.29, 1.24	0.2
Feel supported	0.77	0.47, 1.29	0.3	0.33	0.15, 0.66	0.002
Feel very supported	0.50	0.26, 0.95	0.035	0.31	0.14, 0.69	0.005
Sufficient anaesthesiologist staffing	-	-	0.041	-	-	-
Not sufficient	—	—	-	-	-	-
Some shortcomings, but manageable	0.85	0.52, 1.38	0.5	-	-	-
Sufficient	0.66	0.40, 1.09	0.11	-	-	-
More than sufficient	0.41	0.21, 0.80	0.009	-	-	-
Probability of resigning within 2 years	-	-	<0.001	-	-	0.002
Very unlikely	—	—	-	—	—	-
Unlikely	1.54	0.92, 2.59	0.10	1.50	0.93, 2.40	0.10
Neutral	2.69	1.64, 4.46	<0.001	2.32	1.38, 3.93	0.002
Likely	3.67	2.10, 6.51	<0.001	3.31	1.71, 6.62	<0.001
Very likely	4.00	1.84, 8.95	<0.001	2.53	0.83, 9.63	0.13
Time and finances for social activities	-	-	0.051	-	-	0.011
Not sufficient	—	—	-	—	—	-
Slightly sufficient	0.76	0.46, 1.25	0.3	0.59	0.27, 1.22	0.2
Moderately sufficient	0.51	0.30, 0.87	0.014	0.42	0.19, 0.86	0.023
More than sufficient	0.99	0.30, 3.01	>0.9	0.18	0.05, 0.57	0.005
Neuro anaesthesiology	0.41	0.14, 1.04	0.076	0.46	0.20, 1.02	0.059
General anaesthesiology	-	-	-	1.62	1.01, 2.62	0.046

OR, odds ratio; CI, confidence interval

**Table 5. Summary of Work Life Balance Items:** 

**Items that would improve work life balance**	**Summary statistics**
Improving physical conditions	554 (60.2%)
Flexibility in work hours	474 (51.5%)
Reducing weekly work hours	503 (54.6%)
Increasing the number of vacation days	473 (51.4%)
Having sufficient number of anaesthesiology attendings	282 (30.6%)
Improving workplace culture	534 (58%)
Increased support from leadership	579 (62.9%)
Limiting work related communication outside work hours	218 (23.7%)
Improving salary	736 (79.9%)
Soft skills trainings	246 (26.7%)
Assistance with child/elder care	166 (18%)
Improving efficiency with the electronic medical records	317 (34.4%)
